# SARS-CoV-2 immunity in animal models

**DOI:** 10.1038/s41423-023-01122-w

**Published:** 2024-01-18

**Authors:** Zhao Chen, Yaochang Yuan, Qingtao Hu, Airu Zhu, Fenghua Chen, Shu Li, Xin Guan, Chao Lv, Tian Tang, Yiyun He, Jinling Cheng, Jie Zheng, Xiaoyu Hu, Jingxian Zhao, Jincun Zhao, Jing Sun

**Affiliations:** 1 State Key Laboratory of Respiratory Disease, National Clinical Research Centre for Respiratory Disease, National Centre for Respiratory Medicine, Guangzhou Institute of Respiratory Health, the First Affiliated Hospital of Guangzhou Medical University, Guangzhou Guangdong, 510182 China; 2 GMU-GIBH Joint School of Life Sciences, Guangzhou Medical University, Guangzhou, 510000 China; 3 Guangzhou National Laboratory, Guangzhou Guangdong, 510005 China; 4 Shanghai Institute for Advanced Immunochemical Studies, School of Life Science and Technology, ShanghaiTech University, Shanghai, 201210 China; 5 Institute for Hepatology, National Clinical Research Center for Infectious Disease, Shenzhen Third People’s Hospital, the Second Affiliated Hospital, School of Medicine, Southern University of Science and Technology, Shenzhen, 518005 China

**Keywords:** COVID-19, SARS-CoV-2, animal models, immune response, Immunology, Mechanisms of disease

## Abstract

The COVID-19 pandemic, which was caused by severe acute respiratory syndrome coronavirus 2 (SARS-CoV-2), has become a worldwide health crisis due to its transmissibility. SARS-CoV-2 infection results in severe respiratory illness and can lead to significant complications in affected individuals. These complications encompass symptoms such as coughing, respiratory distress, fever, infectious shock, acute respiratory distress syndrome (ARDS), and even multiple-organ failure. Animal models serve as crucial tools for investigating pathogenic mechanisms, immune responses, immune escape mechanisms, antiviral drug development, and vaccines against SARS-CoV-2. Currently, various animal models for SARS-CoV-2 infection, such as nonhuman primates (NHPs), ferrets, hamsters, and many different mouse models, have been developed. Each model possesses distinctive features and applications. In this review, we elucidate the immune response elicited by SARS-CoV-2 infection in patients and provide an overview of the characteristics of various animal models mainly used for SARS-CoV-2 infection, as well as the corresponding immune responses and applications of these models. A comparative analysis of transcriptomic alterations in the lungs from different animal models revealed that the K18-hACE2 and mouse-adapted virus mouse models exhibited the highest similarity with the deceased COVID-19 patients. Finally, we highlighted the current gaps in related research between animal model studies and clinical investigations, underscoring lingering scientific questions that demand further clarification.

## Introduction

Starting at the end of 2019, the SARS-CoV-2 outbreak first spread in China [1] and then rapidly spread globally, causing a dreadful global pandemic. As of November 30, 2023 [2], SARS-CoV-2 has cumulatively resulted in 772,052,752 confirmed cases and 6,985,278 deaths. In addition to the cases reported by the World Health Organization (WHO), there are still a large number of unconfirmed cases of asymptomatic or mild SARS-CoV-2 infection; therefore, in the real world, the infection rate of SARS-CoV-2 could be much greater than that of other viruses [3]. Although widespread vaccination has made SARS-CoV-2 infection clinically less deadly [4], conducting additional detailed research on this topic is still highly meaningful and will help researchers gain experience for the next pandemic involving other viruses.

To date, a total of seven coronaviruses capable of infecting humans have been identified, including the three highly pathogenic coronaviruses SARS-CoV, MERS-CoV, and SARS-CoV-2 and the four mildly pathogenic coronaviruses HKU1-CoV, 229E-CoV, NL63-CoV, and OC43-CoV [5]. Among them, the three highly pathogenic coronaviruses were successively discovered in the 21st century [6, 7]. SARS-CoV-2 is a single-stranded positive-sense RNA virus that is a beta coronavirus [5], as are SARS-CoV and MERS-CoV. The genome sequence of SARS-CoV-2 shares 79% similarity with that of SARS-CoV and 50% similarity with that of MERS-CoV [8]. Like SARS-CoV, SARS-CoV-2 can encode four structural proteins, namely, the spike protein, the nucleocapsid protein, the membrane protein, and the envelope protein, with the spike protein mainly mediating the virus’s binding to its cellular receptor (angiotensin-converting enzyme 2 (ACE2)) and the process of viral entry into cells [5]. During the pandemic, SARS-CoV-2 has continued to mutate, giving rise to alpha, beta, delta, and omicron variants [9]. Starting from the alpha variant, the spike protein of SARS-CoV-2 acquired the N501Y mutation, leading to increased affinity for ACE2 in mice, thereby enabling direct infection [5]. Subsequently, the N501Y mutation has been found in the beta, delta, and omicron variants [10, 11].

Animal models are vital for studying pathogenic mechanisms, the induction of immune response, immune escape mechanisms, antiviral drugs, and vaccines against SARS-CoV-2. In-depth research on SARS-CoV-2 requires appropriate animal models. Therefore, in this review, we primarily summarize the current progress in animal models of SARS-CoV-2 infection, evaluate the utility of different models in various research directions, summarize the shortcomings of current animal models, and provide suggestions for the development of future animal models.

## Clinical features of COVID-19

### General clinical symptoms of patients

Pneumonia is the most characteristic clinical manifestation of SARS-CoV-2 infection [12, 13]. In addition to pneumonia, COVID-19 can cause a wide spectrum of other clinical symptoms, including conjunctival congestion, nasal congestion, headache, cough, sore throat, sputum production, fatigue, hemoptysis, shortness of breath, nausea or vomiting, diarrhea, myalgia or arthralgia, and chills [1]. During the first wave of the SARS-CoV-2 pandemic in China, several single-center studies and multicenter studies reported that nearly 90% of COVID-19 patients developed fever, and more than 50% of them developed cough, while a small proportion of them developed nausea or vomiting and diarrhea [1, 13]. However, increasing evidence has highlighted the prevalence of asymptomatic infections [14, 15], which account for approximately 40–45% of SARS-CoV-2 infections [16–18].

### Susceptible populations and risk factors

While susceptibility to SARS-CoV-2 is universal, its impact varies significantly among different populations, with certain populations being more predisposed to infection or experiencing more severe manifestations of the illness [19].

The COVID-19 mortality rate is higher in males than in females [1, 20–22]. This difference may be attributed to the apparent stronger innate and adaptive immune responses in females than in males, both in terms of infection and vaccine response [23], as well as multiple other factors, such as sex hormones, genetic factors, social behavioral differences, and comorbidities [24–26].

Age is also an important factor in SARS-CoV-2 infection. Numerous studies have shown that age is an independent risk factor for COVID-19-related mortality [22, 27–29]. Recent research has shown that cellular senescence plays a critical role in regulating cellular changes associated with aging and is a key factor in the excessive inflammation caused by SARS-CoV-2, as the virus triggers and exacerbates the senescence-associated secretory phenotype (SASP), leading to a “cytokine storm” and tissue damage in older individuals [30, 31].

Pregnancy has been reported to be associated with increased disease severity in SARS-CoV-2-infected individuals [32, 33]. The placental inflammation induced by SARS-CoV-2 may account for the increased fetal mortality rather than direct fetal infection [34–38]. At present, whether SARS-CoV-2 has the potential for vertical transmission is not clear. Based on the current data, vertical transmission is not common [39], and further research through animal experiments may be necessary.

The impact of comorbidities on COVID-19 outcomes has been acknowledged since the early stages of the pandemic [1]. According to a cohort study, hypertension (34.3%) was the most prevalent comorbidity, followed by asthma (15.9%) and diabetes (9.9%). Obesity [40] and type 2 diabetes [41] are also independent risk factors for hospitalization and admission to the ICU by COVID-19 patients. Currently, type 2 diabetes in COVID-19 patients may lead to a more robust inflammatory phenotype, leading to inflammatory lung injury [42]. However, type 2 diabetes commonly coexists with obesity [42], and it is difficult to determine which factor determines the ultimate immune phenotype in humans; thus, appropriate animal models are crucial for addressing this issue.

### Receptors and additional host entry factors

The entry of SARS-CoV-2 into cells involves several processes [43]; SARS-CoV-2 initially anchors to the cell surface by binding to its specific receptor ACE2. Subsequently, the spike (S) protein is cleaved into the S1 and S2 subunits by the furin protease, followed by cleavage of the S2’ site either by the cell surface protein transmembrane protease serine (TMPRSS2) or by cathepsins in the endolysosome, particularly cathepsin L. This complete activation of the fusion process enables the virus to enter cells. During this process, SARS-CoV-2 preferentially activates TMPRSS2. If the target cell lacks sufficient TMPRSS2 expression or if the virus-ACE2 complex does not encounter TMPRSS2, the virus bound to ACE2 is internalized via endocytosis mediated by clathrin, leading to late endolysosomal entry, followed by S2′ site cleavage by cathepsins [44, 45]. In addition to ACE2, multiple molecules, including C-type lectins, DC-specific intercellular adhesion molecule-3-grabbing nonintegrin (DC-SIGN) [46], L-SIGN [47, 48], T-cell immunoglobulin and mucin-containing molecule (TIM1) [49], tyrosine-protein kinase receptor UFO (AXL) [50], CD147 [51], and neuropilin 1 [52], have been proposed as alternative receptors for SARS-CoV-2. Due to the widespread expression of receptors and additional host entry factors in various organs of the body, SARS-CoV-2 can cause multiorgan infections. In addition to primary pulmonary and upper respiratory tract infections and respiratory symptoms, COVID-19 patients also exhibit multiorgan dysfunction, with the presence of SARS-CoV-2 RNA has been detected in multiple organ systems [53, 54]. Currently, in-depth research on the tropism of SARS-CoV-2 for different organs is highly valuable, with latent infection with SARS-CoV-2 in the body considered a potential mechanism contributing to long COVID-19 [55].

## Animal models of COVID-19

### Nonhuman primate (NHP) models

Rhesus macaques (RMs) [1, 56], cynomolgus macaques (CMs) [56], and African green monkeys (AGMs) [57–59] have been extensively utilized as models to simulate SARS-CoV-2 infection in humans, which leverages their physiological similarities. NHPs serve as valuable models, replicating mild to moderate manifestations of COVID-19 that are commonly observed in the human population.

One study compared the use of rhesus and cynomolgus macaques as models for investigating COVID-19 infection [56]. Throughout the experiment, no notable weight loss or changes in body temperature were observed. In contrast, other studies have reported that infected rhesus macaques exhibited symptoms such as fever and slight weight loss [60, 61]. In both rhesus and cynomolgus macaques, SARS-CoV-2 replication occurs in the upper and lower respiratory tracts, leading to pulmonary lesions [56]. In cynomolgus macaques, viral loads in the upper respiratory tract (URT) (nasal washes and throat swabs), bronchioalveolar lavage (BAL) and gastrointestinal tract (rectal swabs) were comparable to or greater than those in rhesus macaques [56]. The observed histopathological changes included the thickening of alveolar spaces, damaged alveolar walls, eosinophilic and neutrophilic infiltration, alveolar macrophages, a limited number of lymphocytes, inflammatory cell infiltration, and alveolar type II pneumocyte hyperplasia, as well as edema. The lungs of rhesus macaques displayed pathological changes similar to those observed in cynomolgus macaques, including an increased prominence of bronchial-associated lymphoid tissue (BALT). Therefore, both RMs and CMs are susceptible to SARS-CoV-2 infection, and the utilization of CMs will significantly alleviate the pressure on the limited availability of RMs. After the primary challenge, AGMs exhibited mild and varied clinical signs of disease. Necropsy findings revealed different degrees of pulmonary consolidation accompanied by hyperemia and hemorrhage in the lungs [59]. In addition, SARS-CoV-2 was detected in rectal swabs up to 15 days post infection (dpi), and the virus titer peaked at 2-7 dpi [58, 59]. Prominent features included significant inflammation and coagulopathy evident in both blood and tissues. There was upregulated expression of IFN-stimulated genes (ISGs), as well as increases in IL-6 and IL-8 signaling activation, in the infected lungs and peripheral blood. Moreover, the levels transcripts associated with NK and T cells were decreased [59], which is consistent with what has been observed in human cases. Although a slight increase in antibody concentration was induced after the primary challenge, this increase was enough to clear the virus rapidly after rechallenge with an identical strain [59].

### Ferret models

Ferrets are widely utilized as animal models for researching respiratory viruses that have implications in human health. Ferrets exhibited susceptibility to SARS-CoV-2, as evidenced by the presence of viral RNA in various bodily fluids, including serum, nasal washes, saliva, urine, and feces. Symptoms such as fever and loss of appetite emerged at 10 and 12 dpi after virus inoculation [62, 63]. Examinations conducted at 13 dpi revealed severe lymphoplasmacytic perivasculitis and vasculitis. Additionally, there was an increase in type II pneumocytes, macrophages, and neutrophils in the alveolar septa and lumen. Mild peribronchitis was also observed in the lungs [62, 63]. Kim et al. reported that aged ferrets, in comparison to their younger counterparts, exhibited more severe clinical symptoms, higher viral loads, prolonged nasal virus shedding, and increased inflammatory cell infiltration in the lungs [64]. Ferrets play a pivotal role as effective models for investigating the transmission dynamics of respiratory viruses, such as SARS-CoV-2. According to multiple transmission studies, all previously unexposed contact animals become infected when cohoused with infected counterparts [64–67].

### Hamster models

The Golden Syrian hamster is extensively employed in experimental animal models and has been documented to facilitate the replication of SARS-CoV-2. After intranasal infection with SARS-CoV-2, infected hamsters exhibit moderate but recoverable respiratory disease. Live virus is detectable in the lungs from 1 to 5 dpi [68, 69], and viral RNA is observed in the respiratory system, feces, and kidney. Immunohistochemistry revealed SARS-CoV-2 N protein positivity in olfactory neurons and the duodenum. Histopathological examinations indicate an increase in inflammatory cells and consolidation, covering 5–10% of the lungs at 2 dpi and increasing to 15–35% at 5 dpi [69]. By 7 dpi, consolidation further escalated, covering 30–60% of the lungs, and a decrease in the number of olfactory neurons is observed in the nasal mucosal region [69]. Notably, no apparent histopathological changes were observed in the brain, heart, kidney or liver at 5 dpi [69, 70]. However, various variant of concern (VOC) strains have emerged, and the Omicron virus induces a milder form of the disease in both mice and hamsters [71]. Golden Syrian hamsters, which serve as an animal model for COVID-19, have been extensively used in research focused on antibodies [72] and vaccines [73, 74].

### Mouse models

The spike protein of SARS-CoV-2 exhibits limited affinity for the mouse ACE2 receptor, leading to a reduced capacity for efficient infection in mice. To overcome this barrier, several SARS-CoV-2 mouse infection models have been established. These include the human angiotensin-converting enzyme 2 (hACE2) transgenic mouse model [75, 76], the recombinant virus expressing hACE2 model [77–80], the reverse genetically modified SARS-CoV-2 infection model [81, 82], and a mouse-adapted virus model [83, 84].

#### hACE2 transgenic and knock-in mouse models

Full-length human ACE2 cDNA was inserted into mouse ACE2 Exon 2 under the control of the mouse Ace2 promoter [76]. Viral RNA was detected in the brain, trachea and lung. No obvious clinical signs were observed in the infected mice. In addition, both young and aged mice expressing hACE2 exhibited interstitial pneumonia characterized by inflammatory cell infiltration, thickening of the alveolar septa, and distinct vascular system injuries [76]. Notably, the aged mice exhibited more severe pathological features, including increased lesions in alveolar epithelial cells and focal hemorrhages. In aged hACE2 mice, an increase in the production of cytokines, including eotaxin, G-CSF, IFN-γ, IL-9, and MIP-1b, was observed [76]. However, the response observed in young mice was comparatively weaker than that in older mice [76]. There are other mouse models in which a similar introduction results in mild infection, with the mice showing no apparent symptoms [75].

A transgenic mouse model (HFH4-hACE2 in C3B6 mice) expressing human ACE2 was constructed using a lung ciliated epithelial cell-specific HFH4/FOXJ1 promoter [85]. These mice exhibited elevated hACE2 expression in the lungs, with differential expression noted in various tissues, such as the brain, kidney, liver, and gastrointestinal tract. In the infected mice, notable symptoms included respiratory distress, peri-bronchial and peri-vascular infiltration, and the presence of edema along with hyaline membrane formation. Notably, deceased mice displayed evidence of brain infection, but this finding may not accurately represent the pathogenesis of SARS-CoV-2 in humans [86].

The human ACE2 protein was expressed under the epithelial cell cytokeratin (K18) promoter in K18-hACE2 transgenic mice [87]. hACE2 has been detected not only in epithelial cells that line the respiratory tract but also in various organs, such as the kidney, liver, spleen, and small intestine, and has low or no expression in the brain [88, 89]. Infected K18-hACE2 mice displayed weight loss and lethargy, with fatalities primarily attributed to central nervous system (CNS) dysfunction. By 5 dpi, the lungs exhibited coalescing interstitial pneumonia, characterized by collapsed alveoli with thickened and congested septa [89]. Mononuclear cell infiltration, including that by lymphocytes, macrophages, and plasma cells, was predominantly observed in the peri-vascular region, peri-bronchioles, alveolar septa, and alveolar sacs, as was pulmonary edema [90]. Significant levels of viral RNA and live SARS-CoV-2 were detected in lung homogenates at 2, 4, and 7 dpi [90]. In contrast, lower amounts of viral RNA were observed in the kidney, heart, and spleen, and minimal or no viral RNA was detected in the gastrointestinal tract. SARS-CoV-2 infection resulted in a notable decrease in the number of B cells, CD4^+^ T cells, CD8^+^ T cells, and monocytes in the peripheral blood at 5 dpi, accompanied by a decrease in pulmonary function. At 7 dpi, the infected lung exhibited an increase in proinflammatory cytokines, including IFN-β, IL-6, CXCL10, CXCL9, CCL5, CCL12, TNF, G-CSF, IL-10, IFN-γ, IL-2, CCL2, CCL3, CCL4, and CXCL1, compared to those in noninfected K18-hACE2 mice [90].

In addition, mice exhibited anosmia during the early stages following infection [91]. SARS-CoV-2 can infect the olfactory epithelium of K18-hACE2 mice [91]. SARS-CoV-2 isolates obtained from the brains and lungs of these mice exhibited variable pathogenicity. The viruses isolated from postinfection brain and lung tissues, when separately administered to hACE2 mice, induced lethal infections in the brain and lungs, respectively [92]. SARS-CoV-2 infection of neurons during the early phases results in anosmia, closely resembling the loss of smell observed after COVID-19 infection. Studies suggest a correlation between disease severity and the viral infective dose in K18-hACE2 mice, which may vary with different virus strains and experimental conditions [93].

Consequently, K18-hACE2 mice serve as a valuable model for investigating the pathological mechanisms underlying both mild and severe COVID-19 and evaluating potential therapeutic approaches.

#### Recombinant virus expression in human ACE2-transduced mouse models

Recombinant human ACE2-transduced mouse models, such as those for adenovirus 5 (Ad5) [77, 78], adeno-associated virus (AAV) [94, 95], Venezuelan equine encephalitis Replicons Particles (VRP) [79], and lentivirus [80], were developed. High viral titers and inflammatory cell infiltration were detected in the lung. However, when adeno-associated virus (AAV) was transduced intraventricularly and mice were intraventricularly administered SARS-CoV-2, weight loss and death occurred [95]. The establishment of a virus intranasally or intratracheally transduced model expressing hACE2 in mouse lungs was efficient and rapid, requiring only 2–3 weeks. This model has potential for rapid validation in vaccine, drug, and antibody testing. The limitation of using transduced mouse models lies in their inability to manifest severe disease and the lack of lethality. The expression of hACE2 depends on the tropism of the virus vectors and the route of SARS-CoV-2 administration, which determines whether pulmonary or extrapulmonary disease manifestations occur. These models cannot accurately reflect the physiological expression of ACE2 or the viral infection status.

#### Mouse-adapted virus mouse models

Mouse-adapted strains play a pivotal role in advancing the understanding of pathogenic mechanisms and facilitating drug/vaccine development for highly pathogenic coronaviruses, such as SARS-CoV MA15 [96]-infected WT BALB/c mice and MERS-CoV MA30 [97]-infected hDpp4 KI C57BL/6 mice. Due to the tendency of knockin mice to predominantly exhibit mild infections or succumb to brain infections, researchers have initiated the development of a mouse-adapted strain model for SARS-CoV-2 with the aim of closely reproducing the pathogenesis observed in COVID-19.

A study focused on the adaptation of the original SARS-CoV-2 strain to the lungs of wild-type BALB/c mice, which resulted in the generation of the mouse-adapted strain MASCp6. The presence of viral RNA was detected in various organs, including the lungs, trachea, feces, heart, liver, spleen, and brain tissues. MASCp6 infection in mice did not lead to significant weight loss but induced moderate interstitial pneumonia, with aged mice exhibiting more severe pathological manifestations than younger mice [83]. After continuing the adaptation process for 36 passages, researchers obtained the MASCp36 virus, which was found to be fatal in 9-month-old BALB/c mice. MASCp36 infection caused necrotizing pneumonia and widespread diffuse alveolar damage (DAD), accompanied by a substantial increase in cytokine and chemokine production in the lung parenchyma. Notable factors included IL-6, CCL7, CCL12, CXCL10, CXCL16, CCL3, CXCL1, and CXCL13. Furthermore, MASCp36 infection led to significant cell death, the loss of AT2 cells, and the infiltration of neutrophils and macrophages. The N501Y and Q493H mutations in the receptor binding domain (RBD) of MASCp36 contributed to a high affinity for hACE2. The MASCp36-infected mouse model effectively simulates severe cases of COVID-19 [83, 98].

Another study employed reverse genetics to augment the affinity of the virus for mouse ACE2, resulting in a mouse model directly susceptible to infection in the upper and lower respiratory tracts [81]. Subsequent serial passaging generated MA10, which caused lethality in 25% of 12-month-old BALB/c mice, with decreased lung function, acute lung injury (ALI), ARDS, chronic lung disease, and the development of infection-induced pulmonary fibrosis [82, 99].

Wong et al. employed a recombinant virus with the spike N501Y mutation and passaged it 30 times in mouse lungs to obtain the SARS2-N501YMA30 mouse-adapted strain. This strain exhibited high-level replication in the lungs, with live virus detected only on the second day post infection in the serum, brain, heart, liver, kidneys, spleen, and small intestine. The SARS2-N501Y_MA30_ strain caused lethality in both young BALB/c mice and middle-aged C57BL/6 mice, and induced peripheral blood lymphopenia in middle-aged mice. Increased levels of eicosanoids, specifically prostaglandin D2 (PGD2), and elevated phospholipase (phospholipase A2 group 2D or PLA2G2D) levels during SARS-CoV infection were linked to adverse outcomes in aged mice [100], demonstrating the increased susceptibility of aged or middle-aged mice to SARS-CoV-2 infection [101].

Yan et al. conducted a study involving the preparation of two mouse-adapted SARS-CoV-2 strains, namely, BMA8 in BALB/c mice and C57MA14 in C57BL/6 mice. Both viruses exhibited lethality in young and middle-aged BALB/c mice, but C57MA14 induced lethality in middle-aged C57BL/6 mice, with partial lethality observed in young C57BL/6 mice. The infected mice demonstrated high levels of viral replication in the lungs, elevated inflammatory responses, and lymphopenia [102]. Another mouse-adapted virus, HRB26M, was nonlethal and might be associated with mutation of the adaptogenic strain and the initial strain [103].

The mouse-adapted strain model effectively recapitulates the progression of infection and lung injury repair observed in COVID-19 patients. Underlying conditions or genetic factors are crucial risk factors for severe COVID-19 infections. The limited binding of mouse ACE2 to SARS-CoV-2 limits the study of certain disease models related to underlying conditions. Therefore, the SARS-CoV-2 mouse-adapted strain can be used to directly infect with any wild-type mouse or mouse model under specific conditions, thus allowing for a more comprehensive elucidation of pathogenic mechanisms based on sex, age [101, 104], obesity [105], diabetes, chronic lung disease, coinfection with other respiratory viruses, and secondary bacterial infection (Table 1).

**Table 1:** SARS-CoV-2 mouse-adapted virus mouse models

Animal	Virus strain	Pathology	Lethality	Virus loading in Respiratory tract	Virus loading in other organs	Ref
WT BALB/c WT C57BL/6J	MASCp36	Desquamative epithelial cells and a large area of necrotic alveoli epithelial cells, fused alveoli walls with inflammatory cells infiltration (neutrophils), serious edema, polykaryocytes, hemorrhage, foamy cells, fibrin cluster deposition, and hyaline membrane formation.	Yes, (9-month-old BALB/c mice)	Trachea, Lung	Kidney RNA positive at 2 dpi.	[98]
WT BALB/c	MA10	Thicken alveolar septae, pneumocyte degeneration and necrosis, congestion of small vessels and capillaries, endothelial activation, macrophages and neutrophils infiltration, exudation of proteinaceous fluid and fibrin.	Yes, 25%-90% lethality of 12-month-old BALB/c mice	Lung, nasal turbinate	Not detect	[82, 99]
WT BALB/c WT C57BL/6J	SARS2-N501Y_MA30_	Alveolar edema, perivascular, peribronchial and interstitial infiltration, occasional pulmonary vascular thrombi.	Yes, (9-month-old BALB/c mice)	Lung	Infectious virus was detected in brain, heart, liver, kidney, spleen, intestines at 2 dpi.	[101]
WT BALB/c WT C57BL/6N	BMA8, C57MA14	Gross pulmonary edema, focal hemorrhage, lung bullae, alveolar damage, thicken alveolar septa, inflammatory cell infiltration.	Yes, young and middle-aged BALB/c mice, middle-aged C57BL/6 mice, 100% lethal; young C57BL/6 mice, partial lethal	Lung, nasal cavity	Viral RNA and live virus are detected in blood, brain, heart, liver, kidney, spleen, intestines, at 3 and 5 dpi.	[102]
WT BALB/c WT C57BL/6J	HRB26M	Mild pathological changes in young BALB/c mice: moderate to severe pathological changes in aging adult mice	No	Lung, nasal turbinate	The viral RNA was detected in the heart, liver, kidney, and spleen at 3 dpi.	[103]

#### Long COVID-19 models

Long COVID-19 or postacute sequelae of SARS-CoV-2 infection (PASC) refers to a collection of symptoms that persist for an extended period (often weeks to months) after the acute phase of COVID-19. Symptoms may include fatigue, cognitive difficulties, shortness of breath, joint pain, and other persistent health issues. Long COVID-19 can affect multiple organs, including the respiratory system, cardiovascular system, and neurological system. RMs and AGMs were inoculated with the 2019-nCoV/USA-WA1/2020 strain of SARS-CoV-2, and neuroinflammation, neuron degeneration, apoptosis, neuronal damage, chronic hypoxemia and brain hypoxia were observed in SARS-CoV-2-infected NHPs [106]. Therefore, the NHP model may be used to assess neurological symptoms associated with “long COVID-19”.

In the SARS-CoV-2-infected Golden hamster model, there was a decrease in burying activity compared to that in mock-treated animals, indicating heightened compulsiveness or anxiety-like behaviors. Despite viral clearance, both the olfactory bulb and olfactory epithelium exhibited myeloid and T-cell activation, proinflammatory cytokine production, and an interferon response [68]. Another hamster model study indicated that SARS-CoV-2 infection induced dysregulated alveolar regeneration and subpleural fibrosis [107].

K18-hACE2 mice infected with a low dose of SARS-CoV-2 exhibited characteristic lung fibrosis and increased expression of the proinflammatory kinase B1 receptor (B1R) in the brain. These mice displayed cognitive impairments that were characterized by heightened anxiety and diminished exploratory behavior and attributed to the enduring effects of SARS-CoV-2 infection on brain tissue. Model mice with moderate SARS-CoV-2 infection exhibit increased CCL11 levels and microglial activation, which are associated with neurogenesis impairment and cognitive dysfunction [108]. K18-hACE2 mice may have potential as a model for investigating long COVID-19 in the nervous system [95, 109].

Mouse-adapted (MA10) SARS-CoV-2 induces clinical symptoms of respiratory distress and lung fibrosis in mice [99]. Recently, Gressett et al. reported that MA10 infection induced brain pathology and neuroinflammation in ten-week-old and one-year-old female BALB/cAnNHsd mice at 60 dpi. Pathological changes included a decrease in neurons and an increase in microglia in the hippocampus, which contributes to long-term neurological alterations in a brain region that is crucial for the consolidation and processing of long-term memory [84]. This model holds promise for conducting research on neural inflammation and the recovery of brain function in individuals experiencing persistent cognitive dysfunction associated with “long COVID-19”, thereby facilitating the swift development of innovative therapeutic strategies [84]. In the AAV-hACE2-transduced immunocompetent humanized MISTRG6 mouse model of infection with SARS-CoV-2, there were induced innate and adaptive immune responses for up to 28 dpi. These cells exhibited key features of chronic COVID-19, such as weight loss, lung fibrosis, sustained viral RNA, a macrophage response, continuous expression of interferon-stimulated genes, and T-cell lymphopenia [110].

#### Transmission models

The primary modes of transmission for SARS-CoV-2 include respiratory droplets, contact, and respiratory aerosols. Ferrets serve as a valuable model for investigating the transmission dynamics of respiratory viruses. Despite the absence or mild presentation of clinical symptoms, considerable shedding of SARS-CoV-2 is observed in the respiratory tract. According to numerous transmission studies, all previously unexposed contact animals became infected during cohousing. Direct contact transmission of SARS-CoV-2 in ferrets was effectively prevented by intranasal administration of a fusion inhibitory lipopeptide. This model is well suited for assessing the efficacy of drugs to prevent SARS-CoV-2 transmission [64–67]. Syrian hamsters infected with SARS-CoV-2 can transmit the virus to uninfected hamsters through both direct contact and aerosols. The virus replicates in nasal and lung tissues, causing damage to all cells within the olfactory receptor cell (ORN) lineage [66, 69, 111, 112]. Raccoon dogs, which are members of the canid family, are among the animals whose genetic material has been identified in swabs collected from the Huanan Seafood Wholesale Market in Wuhan, China that is associated with the emergence of the COVID-19 pandemic. Raccoon dogs infected with SARS-CoV-2 exhibited mild clinical symptoms, with viral replication and tissue lesions primarily observed in the nasal conchae. Furthermore, approximately two-thirds of the Raccoon dogs that came into contact were found to be seropositive [113]. Evaluation of the three transmission routes in hACE2-KI C57BL/6 mice revealed that approximately 53.8% of the closely contacted hACE2-KI C57BL/6 mice were seropositive for SARS-CoV-2 antibodies. Additionally, 30% of hACE2 KI C57BL/6 mice were seropositive for SARS-CoV-2 antibodies through exposure to respiratory droplets, and successful aerosol inoculation of hACE2 mice required elevated viral concentrations [114]. Rodriguez et al. demonstrated that neonatal K18-hACE2 mice aged 4-7 days could release the virus and transmit it among siblings within the same litter, resulting in a 100% mortality rate among the exposed pups [115].

## Immunity towards SARS-CoV-2

### Innate immune response in patients

The innate immune system represents the first line of defense against viral invasion and involves not only immune cells but also tissue cells. Ciliated cells in the nasal cavity serve as the initial target for the entry of SARS-CoV-2; these cells allow the virus to propagate to other areas of the respiratory tract through replication and release within these cells [116]. Simultaneously, virus-infected epithelial cells undergo pathological cell death [117], during which viral proteins are released. Subsequently, phagocytic cells at the mucosal site take up both free viral particles and viral proteins. During this process, infected cells and phagocytic cells recognize and bind pathogen-associated molecular patterns through pathogen recognition receptors (such as Melanoma differentiation-associated gene 5 (MDA5) [118], Toll-like receptor 3 (TLR3) [119], and TLR7 [120]), thereby activating downstream pathways related to interferon-regulatory factor 3 (IRF3), nuclear factor-kappa B (NF-κB), and other transcription factors, and generating IFN and inflammatory responses to combat viral infection. Defects in TLR3 and TLR7 have been found to lead to severe COVID-19 [121, 122], highlighting the critical role of pathogen recognition receptor-mediated innate immune responses in controlling COVID-19 infection. A delayed and impaired type I interferon response is considered to be a significant factor that can induce severe cases of COVID-19 [123], possibly due to the presence of autoantibodies against type I interferon [124]. Moreover, excessive levels of proinflammatory cytokines further promotes the infiltration of inflammatory cells [125]. Patients with COVID-19 exhibited increased numbers of neutrophils in the circulation and lungs, which indicate enhanced functionality in NETosis and oxidative bursts [126]. These neutrophils are believed to induce excessive inflammation and promote immune thrombosis, thereby leading to the development of severe COVID-19 [127, 128]. Neutrophils can also be associated with long COVID-19 [129]. In patients previously hospitalized for COVID-19, longitudinal tracking of alternative neutrophil extracellular trap (NET) markers for at least 6 months revealed sustained elevation in the serum concentrations of NE, MPO, and free DNA. While these levels were lower than those in acute COVID-19 patients, they remained higher than those in the non-COVID-19 control group [130]. Moreover, NETs are novel antigens for the adaptive immune system that potentially induce autoantibodies and trigger persistent autoimmune inflammation [129, 131]. The frequency and absolute numbers of blood pDCs and DCs, particularly cDC1s and pDCs, in COVID-19 patients are reduced [132–134]. Furthermore, decreases in HLA-DR and CD86 on DCs may lead to delayed or impaired T-cell responses, thus contributing to the development of severe COVID-19 [134–136]. Alberto Pérez-Gómez et al. reported that the quantity and functional defects of DCs persist seven months after infection [137]. However, little is known about the presence of dendritic cells in the respiratory tract of COVID-19 patients. Research indicates that during SARS-CoV-2 infection, large numbers of monocytes in the peripheral blood may be recruited to the lower respiratory tract via the CCR2-CCL2 axis [138–140], which subsequently differentiate into monocyte-derived macrophages in the lungs. These macrophages can secrete numerous chemokines and inflammatory factors, yet severe pneumonia is unlikely to be attributed to the excessive production of proinflammatory cytokines by these cells [133]. At the site of infection, the function of macrophages extends beyond cytokine secretion to antigen presentation, nd these macrophages providing signals for the sustained activation of specific T cells. In patients with severe COVID-19, impaired antigen presentation by alveolar macrophages can be observed [133] and is potentially associated with compromised T-cell responses. Monocyte-derived macrophages also exhibit a profibrotic phenotype that is closely associated with the profibrotic environment in the lungs during severe COVID-19 [141]. Currently, whether macrophages in the respiratory tract are associated with long-term COVID-19 remains unclear. Natural killer (NK) cells, which are important effector cells within the innate immune system, are capable of directly eliminating virus-infected cells [142]. Previous studies have indicated that upregulated expression of inhibitory receptors, downregulated expression of activating receptors, and an exhausted phenotype in NK cells may be associated with severe COVID-19 [143].

### Innate immune responses in animal models

The pathogenesis of this disease differs in each mouse model. Compared with those of WT mice, mice lacking IFN-I and IFN-II receptors were more susceptible to viral replication and had worsened pulmonary congestion when infected with SARS-CoV-2 [82]. In contrast, K18-hACE2 mice exhibited robust infection accompanied by sustained increases in the production of IFN-I, IFN-II, and IFN-III, which persisted until the virus reached peak replication. This finding underscores the link between IFN expression and the severity of COVID-19 in patients [91, 144]. When WT mice were infected with MA-induced SARS-CoV-2, the increase in IFN production was detrimental because it intensified the pathogenic inflammatory response [145]. Likewise, K18-hACE2 mice infected with SARS-CoV-2 exhibited significant immune cell migration to the lungs; the immune cell population included DCs, monocytes, macrophages, and CD4^+^ and CD8^+^ T cells, as well as significant morbidity and death [91]. Thus, prophylactic and therapeutic IFN therapy protected against SARS-CoV-2 infection in BALB/c mice, as observed with SARS-CoV and MERS-CoV infection [81]. Animals given IFN-III had far less viral proliferation in their lungs [81]. Taken together, these findings showed that early IFN signaling provides protection against SARS-CoV-2 infection, but inadequate IFN induction or extended IFN expression provides only a limited level of protection against viral infection.

Other animal models of infection with SARS-CoV-2, such as those in hamsters, ferrets, and NHPs, exhibit modest disease [146]. IFN-β is not produced in hamsters or ferrets infected with SARS-CoV-2 [147, 148], although the expression of ISG15 was detected after infection and persisted at a high level until 8 dpi, at which point the virus had completely cleared [148]. IFN-α administered prophylactically to hamsters could lower viral titers and ameliorate lung inflammation. Conversely, NHPs showed a greater IFN response to SARS-CoV-2 infection than other animal models when they were infected with the virus, as evidenced by the considerable upregulation of IFN-I and IFN-II expression in BALF and the induction of robust ISG expression in the lungs [149, 150]. Additionally, prophylactic IFN-α therapy decreased lung damage and the viral load after SARS-CoV-2 infection in NHPs, suggesting that the IFN-I response is protective [151]. Overall, these findings suggest that prophylactic IFN-I therapy may decrease the viral load in the lung and transmission, indicating the need for additional research on the potential application of this treatment as a high-risk strategy.

SARS-CoV-2 infection triggers the recruitment of inflammatory cells, including macrophages, monocytes, DCs and neutrophils, to the lung. Unrestrained myeloid immune cell infiltration contributes to pathology in the lung through the excessive secretion of proteases and reactive oxygen species and abnormal cytokine production. Characterizing the driver and involvement of myeloid cell infiltration is critical for developing immunotherapies to help improve immune dysregulation in patients infected with SARS-CoV-2. Animal models are valuable tools that allow us to access tissues at any stage of infection.

In SARS-CoV-2-infected cynomolgus macaques, widespread degranulation of mast cells in the lung coincided with severe damage to the airways and lung-associated vasculature, which are consistent with severe outcomes [152]. Degranulation of mast cells was also found in the airways of *AAV-hACE2*-transduced mice and contributed to severe lung pathology after SARS-CoV-2 infection, consistent with clinical observations in COVID-19 patients [152].

A multiomics analysis of rhesus macaques combined with bulk RNA sequencing (RNA-seq) transcriptomic profiling of BALF and peripheral blood samples, as well as serum proteomics, showed that SARS-CoV-2 infection leads to massive macrophage recruitment and activation in the lung [153]. M1 macrophages and IL-6-, IL-10-, and IFNα-activated macrophages in BAL fluid and peripheral blood were enriched on days 1–4 postchallenge [60, 153]. Cellular trafficking studies in rhesus macaques and African green monkeys showed that during the acute phase of infection, CD16^+^ monocytes migrate rapidly from the blood, leading to CD11b^+^CD16^+^ macrophage accumulation in the lungs. Increased monocyte infiltration and macrophage accumulation in the lung are associated with a higher viral load and worse disease outcomes [154].

Similarly, lymphocytes, macrophages and neutrophils accumulate in the alveolar interstitium and consequently result in thickening of the alveolar walls in hACE2 transgenic mice on day 3 post SARS-CoV-2 infection [75], consistent with the findings in K18-hACE2 transgenic mice, HFH4-hACE2 transgenic mice and CRISPR/Cas9-hACE2 knock-in mice [76, 86, 155].

In a recent study, researchers administered the clinically approved JAK1/JAK2 inhibitor baricitinib to rhesus macaques infected with SARS-CoV-2. The findings revealed rapid and remarkably potent suppression of cytokine and chemokine production by lung macrophages in the treated macaques, which resulted in reduced inflammation, decreased lung infiltration by inflammatory cells, diminished NETosis activity, and limited lung pathology [156].

NK cells traffic to infected sites in lung tissue to help clear viruses in SARS-CoV-infected mice [157]. In the hACE2-transgenic mouse model, the level of NK cells in SARS-CoV-2-infected lungs tended to increase [90], which was consistent with the slight upward trend observed in the BALF of COVID-19 patients. However, determining the features and function of NK cells during SARS-CoV-2 infection need additional detailed studies.

These studies provide evidence that innate immune cell function may be an important driver of COVID-19, and modulation of this process could be an effective immunotherapy for disease treatment. However, further studies are needed to explore when and how to modulate myeloid cell function during infection.

### The adaptive immune response in patients

The humoral immune response involves the production of neutralizing antibodies by B cells, which directly exert antiviral effects or generate binding antibodies that mediate antibody-dependent cellular cytotoxicity (ADCC), antibody-dependent cellular phagocytosis (ADCP), and complement-dependent cytotoxicity (CDC), as well as the functions of antigen presentation and cytokine secretion by B cells. Neutralizing antibodies were induced at high levels approximately 10-15 days after onset in patients with severe and mild infection. While both patients with severe and mild infection exhibited similar kinetics of the neutralizing antibody response, the magnitude of the response was positively correlated with disease severity [158]. Moreover, the clonal expansion of B cells and the proportion of plasma cells are both greater in patients with severe COVID-19 than in patients with mild disease [124, 159]. Subsequent studies have shown that elevated levels of antibodies in patients with severe COVID-19 may not correlate with the resolution of the disease [160]. Anti-S IgG may mediate microvascular thrombosis through complement activation [161] or enhance viral entry through the ADE mechanism [162]. In addition, increased viral antigen levels in patients often result in elevated antibody titers. During infection, the levels of IgG antibodies remain relatively stable for up to 5 months [163], which correlates with a significant decrease in the risk of reinfection [164]. While the antibody response in peripheral blood has been extensively studied, reports on the antibody response at the site of infection are scarce. The specific role played by antibodies at the site of infection remains unresolved, necessitating further mechanistic research.

The cellular immune response mainly refers to the specific T-cell response. Virus-specific T cells play a crucial role in combating viral infections [165, 166]. After recognizing specific antigens, they can enhance the antiviral activity of other cells by secreting IFN-γ, regulating the activation and antigen presentation functions of other immune cells, or directly killing infected target cells to exert antiviral effects [167]. Several publications have focused on investigating SARS-CoV-2-specific T-cell responses in the peripheral blood of COVID-19 patients [168–171], with findings suggesting that the rapid induction of virus-specific T cells in peripheral blood may accelerate viral clearance and control pathological damage [171]. Additionally, research has indicated that the functionality of virus-specific T cells in peripheral blood is greater in asymptomatic COVID-19 patients than in symptomatic individuals [169]. These insights underscore the significance of virus-specific T-cell responses in combating COVID-19 infections. Given that the respiratory tract is the primary site that is directly targeted by SARS-CoV-2 [172], further detailed investigations into virus-specific T cells within this region are essential, as they are at the forefront of the fight against viral infections. To date, only a few studies have described the specific T-cell responses in the airways, including the upper and lower respiratory tracts. They found that specific T cells in the airways of COVID-19 patients have strong cytokine-secreting capabilities [173], and long-lived SARS-CoV-2-specific tissue-resident T cells could be induced by SARS-CoV-2 infection [174, 175], which may help prevent future infections. However, there are still several unresolved questions, such as how specific T cells in the airways are regulated during infection, how they function, and how they interact with other cells. Addressing these questions will contribute to the development of future immunotherapy methods and vaccines. Nevertheless, studying these questions in humans is very complex, as there are many confounding factors, and complex issues need to be simplified by relying on animal models.

### Adaptive immune response in animal models

Humoral immune responses against SARS-CoV-2 infection have been described in animal models. In mouse models and hamster models, both virus-specific and neutralizing antibodies were detected within 7 dpi [94, 176]. In addition, in NHP models infected with SARS-CoV-2, virus-specific and neutralizing antibodies developed by 10 dpi, and the titers of antibodies peaked between 15 and 20 dpi [58, 59], mirroring the immune response observed in COVID-19 patients. Furthermore, several other studies have reported that virus-specific antibodies could protect NHPs from SARS-CoV-2 reinfection [149, 177]. In a study involving K18-hACE2 mice, SARS-CoV-2 infection was found to trigger the formation of B-cell clusters that accumulated in the peribronchial areas. Remarkably, even low doses of SARS-CoV-2 infection were sufficient to generate genuine and bystander subsets of lung-resident memory B cells (MBCs), even in asymptomatic animals without apparent weight loss. This finding suggested that asymptomatic individuals are capable of mounting memory responses to SARS-CoV-2 [178].

Numerous lines of evidence from animal models suggest the importance of understanding human SARS-CoV-2-specific T cells. An investigation of T-cell reactions to SARS-CoV and MERS-CoV infection revealed that IFN-γ-induced protective immunity in mice is mediated by airway CD4^+^ T cells [165]. In addition, virus-specific memory CD8^+^ T cells could prevent lethal SARS-CoV infection in mice [179]. These findings imply that controlling SARS-CoV-2 infection may depend on the cellular immune response. The primary problem with SARS-CoV-2 mouse models is that they swiftly lead to virus clearance, rehabilitation or death, which reduces the amount of time T cells can provide defense. In a mouse model of SARS-CoV-2 infection, systemic or lung-resident memory CD4^+^ and CD8^+^ T cells could offer effective protection in the absence of neutralizing antibodies [180]. Through tissue-resident memory T cells, immunization with a single CD8^+^ T-cell epitope can safeguard mice against SARS-CoV-2 infection in the absence of neutralizing antibodies [181]. Another interesting question is the role played by IL-10-producing SARS-CoV-2-specific T cells. A study in human populations has indicated that higher levels of IL-10 in plasma may prevent the onset of symptoms in individuals infected with SARS-CoV-2 [169]. Previous research in mouse models of infection with SARS-CoV has shown that while IL-10 does not affect viral clearance kinetics, it provides protective effects by inhibiting excessive pathological damage [165]. Similarly, IL-10-producing SARS-CoV-2-specific T cells exist in SARS-CoV-2 infection [180], but whether they mediate protective immune effects needs to be further elucidated in a mouse model.

Numerous studies have demonstrated the roles of CD4^+^ and CD8^+^ T cells in protective immunity in NHP models. Comprehensive analyses of CD4^+^ T-cell responses using the NHP model verified that SARS-CoV-2 could elicit a strong CD4^+^ T follicular helper cell response in the germinal center [182]. The same study also showed that CD4^+^ T cells were predominantly involved in Th1-biased responses [182]. Furthermore, in convalescent rhesus macaques, depletion of CD8^+^ T cells decreased protection against rechallenge [166]. These findings were extended in later research on vaccination-mediated defense against Omicron variants, demonstrating the association between reduced viral loads following SARS-CoV-2 challenge and CD8^+^ T-cell frequencies [183]. A study employing intranasal vaccination to produce viral nonspike antigens also revealed the function of CD8^+^ T cells in vaccine-induced protection in rhesus macaques [184]. In summary, a number of lines of research using various animal models have demonstrated the importance of T-cell responses in defending against SARS-CoV-2 infection.

### COVID-19 vaccines and antiviral therapeutics

Since the COVID-19 outbreak, several therapeutic approaches, including the use of vaccines, antibodies, and drugs, have been investigated for COVID-19 treatment to lessen the impact of SARS-CoV-2 on general public health and the economy. Animal models are crucial resources in the development of these treatment approaches.

Mouse models have been the initial choice for the in vivo evaluation of vaccine candidates and antiviral therapeutics for SARS-CoV-2. Compared to other experimental animal models, mice have various advantages, including their small size, low cost, capacity to reproduce quickly to reach large group numbers, and the accessibility of research tools. A variety of COVID-19 vaccine candidates, such as mRNA vaccines (mRNA-1273), adenoviral vector vaccines (ChAdOx1 nCoV-19 and Ad5-nCoV), recombinant subunit vaccines (NVX-CoV2373 and RBD-Fc-based COVID-19), and inactivated vaccines (BBIBP-CorV and PiCoVacc), have been tested in mice. These vaccine candidates provided mice with protective immune responses [185–188] and prevented them from contracting SARS-CoV-2 infection [187, 189, 190]. Remdesivir, an antiviral drug licensed for use in an emergency setting to treat COVID-19, has the potential to lower the lung viral load in mice [191] and was additionally identified in SARS-CoV-2-infected adenovirus-transduced mice and BALB/c mice infected with mouse-adapted SARS-CoV-2 [103]. PEG-IFN-l1a, a phase 3-ready therapy for hepatitis delta virus, diminished the amount of SARS-CoV-2 replication in the lungs of Hfh4-ACE2 transgenic mice [81]. Monoclonal antibodies (mAbs) can be used to generate passive immunity, which is a potential strategy for preventing emerging viral infections. In aged BALB/c mice, neutralizing mAbs from COVID-19 survivors could lower the viral loads of SARS-CoV-2 [192], prevent dramatic weight loss caused by SARS-CoV-2, and reduce viral loads in other mouse models [193]. Similarly, recombinant SARS-CoV-2 RBD- and S protein-immunized mouse-derived neutralizing mAbs successfully reduced viral shedding and clinical symptoms in hACE2-transduced mice [77, 194]. Although the pathogenesis and host immune responses of SARS-CoV-2 in humans may not be entirely reflected in genetically modified mice or mouse-adapted SARS-CoV-2 strains, these mouse models can still be useful for preliminary analyses and large-scale investigations of vaccine candidates and antiviral therapies.

The use of hamster models has been widespread in research on a variety of viral diseases [195]. Model hamsters are very susceptible to SARS-CoV-2 infection, have a rapid reproduction rate, are tiny, have a pathophysiology similar to humans, and have been utilized in a number of preclinical efficacy trials for antiviral treatments and vaccine candidates. Hamster models have proven to be very helpful in assessing vaccine candidates. Hamsters vaccinated with an adenovirus serotype 26 vector-based vaccine that expressed a stabilized SARS-CoV-2 spike protein generated neutralizing antibodies and protected the animals against severe clinical illness [74]. Similarly, an NDV vectored vaccine expressing a membrane-anchored spike protein of SARS-CoV-2 (NDV-S)-inoculated hamsters showed decreased lung virus titers with decreased body weight loss after SARS-CoV-2 challenge [196]. In SARS-CoV-2-infected Syrian hamsters, conventional or high-dose hydroxychloroquine did not diminish viral shedding or demonstrate any therapeutic advantages [197], and favipiravir reduced viral shedding and transmission only at high doses [197]. In a preclinical study using a Syrian hamster model, a single prophylactic dose of drug (CTC-445.2d) was found to be protective against SARS-CoV-2 infection [198]. Furthermore, neutralizing antibodies that target the RBD or spike protein administered prophylactically or therapeutically successfully limited viral shedding in hamster models [199–202].

Ferret models are often used to study the pathogenesis and transmission of influenza viruses because their receptor distribution and clinical course of illness are comparable to those of humans [203]. In early 2020, it was discovered that humans and ferrets have similar ACE2 sequences that are essential for binding viruses, suggesting that ferrets could serve as an animal model for SARS-CoV-2 infection [204]. Due to their high vulnerability to SARS-CoV-2, ferret models are used to assess the effectiveness of antiviral treatments. Model ferrets were used to assess the effectiveness of repurposed drugs and vaccine candidates [205]. MK-4482/EIDD-2801, which is a ribonucleoside analog inhibitor originally used to treat influenza viruses, protected against SARS-CoV-2 infection via oral administration [206]. Additionally, ferrets vaccinated intramuscularly or intranasally with an adenovirus vectored vaccine (Ad5-nCoV) could inhibit viral replication in the URT after being challenged with SARS-CoV-2 [189].

Because NHP models resemble humans in both physiology and phylogeny, they are frequently regarded as the gold standard model for studying emerging viruses [207]. Among the 14 mammalian species, the ACE2 gene of rhesus macaques shares 23 critical residues with hACE2 in the region of the protein that makes close contact with the RBD of the SARS-CoV-2 spike protein and has the greatest receptor activity [208]. The development of COVID-19 vaccines and antiviral therapies in NHPs has attracted significant attention. However, hydroxychloroquine did not demonstrate prophylactic or therapeutic protection in rhesus or cynomolgus macaque models. Moreover, remdesivir has been shown to ameliorate SARS-CoV-2-induced lung damage and lower the viral load in a rhesus macaque model [209]. Neutralizing antibodies that target the spike protein could provide therapeutic activity and protection against SARS-CoV-2 infection in NHP models [202, 210]. In regard to preclinical research, NHP models are advantageous since their immune responses after SARS-CoV-2 infection closely resemble those of key COVID-19 infections in humans [154]. Therefore, scientists evaluating different COVID-19 vaccine platforms, including adenovirus vectored, mRNA, inactivated, and subunit vaccines, have utilized NHP models to evaluate the safety, immunogenicity, and effectiveness of protection [211–213] (Table 2).

**Table 2:** The application of animal models for SARS-CoV-2

Animal models	Advantages	Drugs	NAbs	Vaccines	Innate immunity	Adaptive immunity		Long COVID
						B cells	T cells	
Adenovirus transduced hACE2 mouse	Easily handling, small size, low cost, rapid breeding, availability of research tools	√	√	√	√	√	√	√
Transgenic mouse expressing hACE2		√	√	√	√	√	√	
Mouse Adapted SARS-CoV-2			√	√	√	√	√	√
Hamster	Highly susceptible to SARS-CoV-2, natural ACE2 permissiveness and tissue distribution	√	√	√	√	√		√
Ferret	Relatively small size, highly susceptible to SARS-CoV-2, transmission via direct or indirect contact	√	√	√	√			
Rhesus macaque	Physiologically closest in similarity to humans, timeline of COVID19 clinical symptoms and seroconversion like humans	√	√	√	√	√	√	√
Cynomolgus macaque		√	√	√		√		√
African green monkey			√	√				√

*NAbs* Neutralizing Antibodies

The check mark indicates positive results

## Suitability of animal models for investigating COVID-19: a multi-omics perspective

Currently, a diverse array of small and large animal models, such as the mouse, hamster, ferret, rhesus macaques, and African green monkey models, have become available for studying COVID-19. An imperative consideration is the comprehensive evaluation of these models to determine their fidelity in recapitulating the complexities of COVID-19. Leveraging cutting-edge multiomics technologies, encompassing transcriptomics, proteomics, metabolomics, and epigenetics, will facilitate a panoramic assessment of these animal models.

**Fig. 1:**
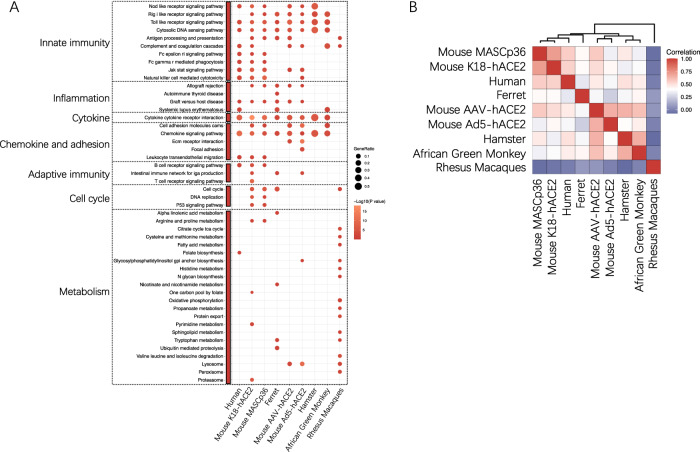
Transcriptomic comparison of COVID-19 patients and animal models. **A** KEGG enrichment analysis of genes with upregulated expression in COVID-19 patients and animal models. Differentially expressed genes (DEGs) were computed for each comparison against their respective controls. Specifically, the genes with upregulated expression were subjected to Kyoto Encyclopedia of Genes and Genomes (KEGG) enrichment analysis using clusterProfiler [216]. **B** For the purpose of similarity comparison, the KEGG enrichment results were dichotomized based on significance (P ≤ 0.05 represented as 1, and P > 0.05 represented 0). Subsequently, Pearson correlation coefficients were calculated for the dichotomized matrix and visualized using ggplot2

Here, our initial exploration entailed comparing transcriptomic alterations in the lung tissue of various animal models with postmortem data from COVID-19 patients. The data sources are presented in Table 3. Significant enrichment of key pathways associated with the innate immune response, inflammation, cytokine‒cytokine receptor interactions, and chemokine and adhesion pathways was observed across multiple animal models, mirroring the signatures observed in human COVID-19 patients (Fig. 1a). The K18-hACE2 mouse model and mouse-adapted virus MASCp36 mouse model, both of which progressed to severe disease phenotypes, exhibited the highest similarity in enriched KEGG pathways with severe cases of human COVID-19 (Fig. 1b). The lack of omics data from lung tissues of mild COVID-19 patients presents a challenge in comparing mild infection models.

**Table 3:** The data sources used for transcriptomic analysis

Model	Model severity	Tissue	Sampling timepoint	Bulk RNAseq data source
Human	Severe	Lung	post-mortem	[147]
Rhesus Macaques	Mild	Lung	14dpi	[214]
African Green Monkey	Mild	BALF	3dpi	[59]
Hamster	Mild	Lung	3dpi	[68]
Ferret	Mild	Lung	2dpi	[64]
Mouse MASCp36	Severe	Lung	4dpi	[98]
Mouse K18-hACE2	Severe	Lung	6dpi	[215]
Mouse Ad5-hACE2	Mild	Lung	2dpi	[78]
Mouse AAV-hACE2	Mild	Lung	2dpi	[94]

## Conclusions and perspectives

In-depth research on SARS-CoV-2 infection requires the use of appropriate animal models, which are essential for understanding the pathogenesis and immune responses of this disease and will contribute to the development of antiviral drugs and vaccines. Here, we provide a comprehensive summary of diverse animal models used for SARS-CoV-2 infection, encompassing NHPs, ferrets, hamsters, and a variety of mouse models, each with unique attributes and applications. Moreover, in this review, we delineate the immune response evoked by SARS-CoV-2 infection in human patients and present an overview of the immune response characteristics in different animal models of SARS-CoV-2 infection. Finally, we compared the pulmonary tissue transcriptomic data from diverse animal models with those from deceased COVID-19 patients. Our analysis revealed that the severe K18-hACE2 mouse model and the mouse-adapted strain MASCp36-infected mouse model demonstrated the greatest resemblance in terms of transcriptome data to that of deceased COVID-19 patients. However, there are still several research gaps.

1. To date, most animal models, with the exception of mouse models, mimic the mild form of COVID-19. However, due to the complexity of the human immune system compared to murine models, developing nonhuman primate models of severe pneumonia is necessary to better elucidate the immunoregulatory mechanisms involved in severe COVID-19 pneumonia.
2. The presence of local immune agents in the lungs and respiratory mucosal immunity, as well as the interaction of these agents with the systemic immune response, should be thoroughly investigated in animal models.
3. Animal models should be utilized to investigate the threshold of immune protection, specifically the level of immune response required to protect against SARS-CoV-2 infection, encompassing both the humoral immune response and the T-cell response.
4. Emphasizing the current gap in multiomics research related to COVID-19 and animal models is crucial. A more comprehensive investigation of multiomics data types, including bulk and single-cell datasets and those covering a wider range of tissue types, is necessary. Conducting thorough analyses is essential for improving model selection criteria and interpreting results, thereby ultimately advancing our understanding of the immune response to COVID-19.
5. Patients with preexisting comorbidities exhibit heightened symptom severity upon infection, yet our understanding of the mechanisms by which comorbidities affect SARS-CoV-2 infection is limited. Therefore, developing animal models that recapitulate the influence of comorbidities on SARS-CoV-2 infection is imperative.
6. However, further research using animal models is needed to investigate the mechanisms of age-related severe COVID-19 and to develop corresponding interventions.
7. Investigating the potential risk of vertical transmission of SARS-CoV-2 in animal models, as well as the effects of maternal vaccination during pregnancy on both the mother and fetus, is imperative.
8. Investigating the long-term effects of prior COVID-19 infection on the immune system in animal models, including potential induction of autoimmunity and allergic reactions and impact on host resistance to other pathogen infections, is essential.
